# Neurobiological Link between Stress and Gaming: A Scoping Review

**DOI:** 10.3390/jcm12093113

**Published:** 2023-04-25

**Authors:** Grace Y. Wang, Dovile Simkute, Inga Griskova-Bulanova

**Affiliations:** 1 School of Psychology and Wellbeing, University of Southern Queensland, Toowoomba, QLD 4350, Australia; 2 Centre of Health Research, University of Southern Queensland, Toowoomba, QLD 4350, Australia; 3 Institute of Biosciences, Life Sciences Centre, Vilnius University, 10257 Vilnius, Lithuania

**Keywords:** video gaming, addiction, stress, physiological response, inflammation

## Abstract

Research on video gaming has been challenged by the way to properly measure individual play experience as a continuum, and current research primarily focuses on persons with gaming disorder based on the diagnostic criteria established in relation to substance use and gambling. To better capture the complexity and dynamic experience of gaming, an understanding of brain functional changes related to gaming is necessary. Based on the proinflammatory hypothesis of addiction, this scoping review was aiming to (1) survey the literature published since 2012 to determine how data pertinent to the measurement of stress response had been reported in video gaming studies and (2) clarify the link between gaming and stress response. Eleven studies were included in this review, and the results suggest that gaming could stimulate a stress-like physiological response, and the direction of this response is influenced by an individual’s biological profile, history of gaming, and gaming content. Our findings highlight the need for future investigation of the stress-behaviour correlation in the context of gaming, and this will assist in understanding the biological mechanisms underlying game addiction and inform the potential targets for addiction-related proinflammatory research.

## 1. Introduction

Video games have progressed significantly since the release of Pong, the first arcade game, in 1972 [1]. Since then, video games have evolved from black and white, two-dimensional graphics into the current state of games with amazing 3D graphics and hooking storylines, which can be addicting. Recent advancements in technology have made games extremely accessible on devices such as tablets, smartphones, computers, smart televisions, virtual reality, and gaming consoles [2].

Currently, it is difficult to estimate the prevalence of problematic gamers worldwide due to the lack of a clear definition and internationally agreed-upon psychometric measures in problematic gaming [3]. Research on a United States and Singaporean national representative sample of adolescents found problematic game use at approximately 9% [4], 0.4% in Poland, and 4.7% in Indonesia [5]. In a Norwegian sample of 3389 [6] and a Dutch sample of 902 [7], it was found that only 1.4% were addicted gamers in both studies. However, the low prevalence rates in both studies could be a result of the age characteristics of the sample, as they included seniors aged up to 81 years old who may be less interested in gaming. Moreover, 67% of New Zealand’s population are gamers, with 9% being problematic gamers [8]. It was also found that 53% of New Zealand gamers are men, and 73% are adults aged 18 years or older [8].

The reasons for game playing are fluid and vary between individuals, as some games serve as a method to cope with stress, while others are played for entertainment, out of boredom, in search of challenges, or as a way to pass the time [9]. The consequence of poor coping methods and the advancement of technology has resulted in a constantly growing number of gamers who are addicted to having the ability to play games portably, which allows them to spend time gaming at any time and place [9,10]. This is an issue because there is no restriction on the location of gaming, which allows individuals to game at work, school, while driving, or in public places. As a result, areas such as work and school performances are affected, putting oneself and others in danger.

At present, research on video gaming has been challenged by the lack of ways to properly measure individual play experiences. Attention has largely been addressed to those who are defined as problem gamers or persons with gaming disorder based on the diagnostic criteria established in relation to substance use and gambling [11]. However, the reality is that “problem” gaming and healthy engagement with gaming as a leisure activity can be challenging to differentiate. Positive and negative effects of gaming could appear simultaneously and be shaped by sociocultural factors such as social isolation and loneliness [12,13]. Thus, there is a need to shift from the traditional way of assessing gaming in binary forms of “disease” or “health” to a new approach that defines gaming as a continuum; this would help to capture the complexity and dynamic experience of gaming, leading to great potential to avert disease before onset [12]. 

A framework linking the adverse effects of gaming with compromised molecular physiological conditions has been proposed [12] and is in line with the proinflammatory hypothesis of addiction, suggesting modulating pro-inflammatory molecules could lead to brain functional and behavioural changes contributing to addiction [14,15]. One of the main routes to locate the innate immune system implicated in addiction is through the nuclear factor kappa-light-chain-enhancer of the activated B cell (NF-kB) signalling pathway [16]. This pathway increases the release of proinflammatory cytokines such as Interleukin-1 beta (IL-1β) and tumour necrosis factor-alpha (TNF-α), influencing the neurobiological processes involved in the development of addiction [17]. Furthermore, involvements of other cells, such as IL-6, IL-1β, IL-2 IFN-γ, and C-reactive protein (CRP), in addition, have also been reported [16]. Importantly, the increased release of proinflammatory cytokines frequently follows the chronic stress response, stimulating the Hypothalamic-Pituitary-Adrenal (HPA) axis [18]. Stress leads to an increase in HPA axis activity to a stressor by initiating the release of corticotrophin-releasing factor (CRF) at the hypothalamus [19]. CRF secretion initiates the release of adrenocorticotropic hormone (ACTH) from the pituitary and glucocorticoids (GC), such as cortisol, from the adrenal gland [19]. GCs not only mediate the stress response but also have widespread homeostatic control over the body, including the immune system with potent immune-suppressive and anti-inflammatory effects [20]. Acute and chronic stress differ at HPA axis activation, with chronic stress leading to the hypersecretion of CRF and GCs and hyposensitivity to feedback inhibition regulated by cortisol [18]. It has been shown that reduced glucocorticoid responsiveness leads to increased concentrations of proinflammatory cytokines, plays a role in major depression [21], and is involved in the development of addiction [22].

Although a recent review suggests an association between video game playing time and physical health deterioration indicators, including BMI and general health status [23], the implication of this association for the immune system is unclear. The degree of psychological and immune reactivity in response to acute stress has been shown to predict depression symptoms and susceptibility to addiction, suggesting the potential of using these measures for assessing individual vulnerability to mental disorders [24]. Given that stress relief has been reported by game players as one of the main motivations for gaming, the aim of this scoping review was to survey the literature to determine how data pertinent to the measurement of stress response had been reported in video gaming studies and clarify the link between gaming and stress response. We believe that investigating the stress–behaviour correlation will assist in understanding the biological mechanisms underlying video game addiction and inform the potential targets for addiction-related proinflammatory research. To achieve the goals of the present review, three research questions were proposed for guidance, including: (1) Who are the research participants examined in the gaming-related stress research? (2) What measurements are used to measure gaming-related stress? (3) What is the neurobiological evidence in stress-related gaming research?

## 2. Methods

A scoping review methodology defined by Arskey and O’Malle [25] was used. Relevant articles were sought from PubMed, Scopus, and EBSCO (databases at EBSCOhost comprised of APA PsycArticles, APA PsycInfo, MEDLINE, and MEDLINE Ultimate) between 2002 and 15 October 2022, using the following search terms to search the databases (all in titles and abstracts): (gaming OR game) AND (stress OR immun*). As the literature search was not conducted on a systematic basis, the evidence reviewed may not be exhaustive.

### 2.1. Study Selection

The criteria for article inclusion in the review was the employment of neurobiological methods such as electroencephalography, electrocardiography, and other cardiovascular function parameters, cortisol, and/or other hormones/metabolites measures to investigate stress or immune function, used to investigate the gaming behaviour of adolescents and adult participants. Only articles in English published in the last 10 years (between 2012 and 2022) were selected. Articles not available in the full text or published in a peer-reviewed format, as well as reviews, meta-analyses, opinions, and editorials, were excluded. Although the criteria for inclusion was the investigation of gaming behaviour, studies investigating e-sports or watching the games played by others were excluded, as the competition level of e-sports made it unique from general gaming.

**Figure 1:**
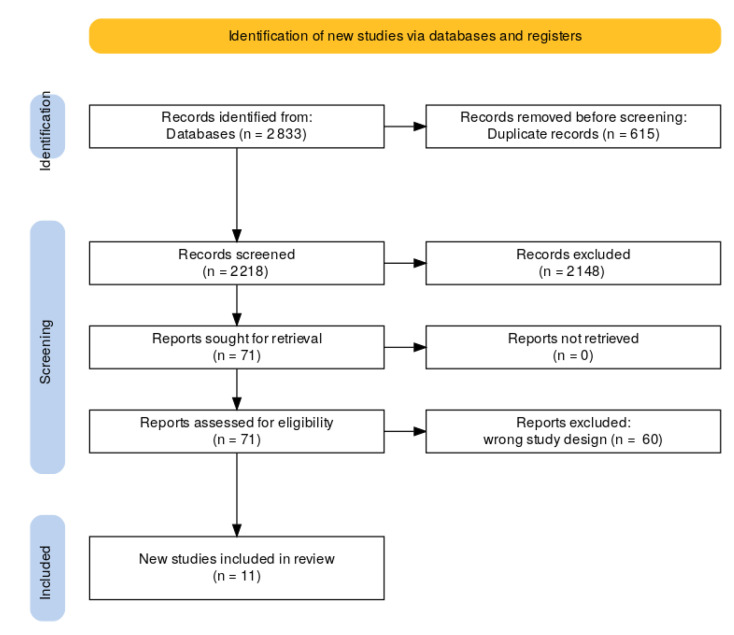
PRISMA flow diagram of the literature search.

The databases were scanned based on the search terms, and the search results were imported to a systematic review web tool, Rayyan [26]. Duplicates were deleted, and the remaining articles were screened by titles and abstracts. Eleven articles met the criteria and were included in this review. The illustration of the study selection process is presented in Figure 1 using the PRISMA flow diagram [27].

### 2.2. Synthesis of Results

The articles were subsequently discussed through consensus between authors (Table 1).

### 2.3. Research Participants and Study Design

Study sample sizes ranged from 23 to 148 participants, while group sizes varied from 11 to 50 individuals per group. Only male participants were enrolled in seven out of 11 studies [28,29,30,31,32,33,34]. While three studies enrolled adolescents as well—two with a defined age range from 15 to 25 years [31,33] and one from 16 to 29 years [32], the rest involved adult participants only.

Three studies included IGD groups and healthy participants as controls [31,33,34], and one study additionally included individuals with alcohol use disorder [35]. Different types or modes of video games for participants’ allocation into different groups were used in three studies [29,36,37]. One study compared high vs. low gaming involvement groups [38], two studies compared experimental groups vs. control groups [30,32], and only one study investigated the experimental group solely [29].

Of the 11 studies, two employed a comparative cross-sectional design, which compared gamers with control/comparison groups [33,35], and the rest employed a repeat measure design, where the same gamers were assessed at multiple time points [28,29,30,31,32,34,36,37].

**Table 1:** Overview of the studies included in this review.

#	Authors	Main Aim Of The Study	Participants (N = Total Number, Gender, Age [M ± SD])	Methods	Main Physiological Results
1	(Aliyari et al., 2015) [28]	To study and analyze the changes in cognitive and hormonal changes created in brain waves while playing a double tournament game of FIFA 2015.	N = 32; male only Average age: 20	Questionnaires: personal details and questions about gaming Double tournament game of FIFA 2015 football match Saliva samples [cortisol] (before, after the game) The PASAT test (before, after the game) Wireless EEG (during the game)	Salivary cortisol concentration: decreased after the game (vs. before the game) PASAT test (before and after the game): Mental health (number of correct answers)—no significant changes; Response time—increased after; Sustained attention (longest chain of correct responses)—increased after (NS); Mental fatigue (longest chain of wrong responses)—no significant changes. Changes in the brain waves: During high activity, the power in lower frequency bands increased. High activity during playing decreased the power in lower frequencies in occipital lobe and in higher frequencies in frontal electrodes.
2	(Aliyari et al., 2018) [29]	To examine the different types of stress with regard to the style of video games.	N = 80; male only 18–30 years 20 Runner game group 20 Excitement game group 20 Fear game group 20 Puzzle game group	Questionnaires: personal details and questions about gaming Four types of video games (Runner game, Excitement game, Fear game, and Puzzle game). Each group played one type of the games. Saliva samples [cortisol, α-amylase] (before, after the game) Wireless EEG (during the game)	Salivary α-amylase concentration: decreased after playing the Puzzle game, increased after playing the Runner game, the Fear game and the Excitement game. Salivary cortisol concentration: decreased after playing the Puzzle game, increased after playing the Runner game, the Fear game, and the Excitement game. Changes in the brain waves: the ratio of the alpha power of the frontal left hemisphere to the frontal right hemisphere was higher in fear game.
3	(Aliyari et al., 2021) [30]	To evaluate the short-term effects of the brain teaser game on players	N = 40; male only Average age: 20 20 experimental group 20 control group	Questionnaires: personal details and questions about gaming A brain teaser game (only for experimental group, no game for control group) The PASAT test (before, after the game) Saliva samples [cortisol, α-amylase] (before, after the game) Wireless EEG (before, during and after the game)	PASAT test (before and after the game): Mental health (number of correct answers)—increased after (experimental group); Sustained attention (longest chain of correct responses) increased after (experimental group). Salivary α-amylase and cortisol concentration: higher after the intervention (experimental group; no changes in control group) Changes in the brain waves: the ratio of the activity of the right hemisphere relative to the left hemisphere was greater after gaming (no changes in control group).
4	(Jang et al., 2022) [35]	To investigate the serum concentrations of KYN pathway metabolites—including TRP, 5-HT, KYN, and KYNA—as well as stress levels, resilience, and neurocognitive functions in young adults with AUD and IGD and HCs.	N = 99; males 85, females 14 18–35 years 30 AUD [27.2 ± 5.1] 34 IGD [23.5 ± 4.2] 35 HCs [24.6 ± 2.9]	A clinical interview by an experienced psychiatrist based on the criteria of the DSM-5 Questionnaires: AUDIT, Y-IAT, WAIS-IV, PWI, CD-RISC, and BDI, BAI Executive Function assessment: K-CWST, TMT, and CANTAB to assess SWM and SSP Analysis of Kynurenine Pathway Metabolites: TRP, 5-HT, KYN, and KYNA levels in serum samples	AUD and IGD groups: higher stress levels and lower resilience levels than HC group. AUD group: higher scores for SWM strategy than IGD and HCs. Increases in KYN level and the KYN/TRP ratio; decreases in KYNA and the KYNA/KYN ratio compared to HCs; KYN metabolites more extensively associated with stress levels and executive dysfunction than in the IGD; relationship between stress level and KYN increased with higher resilience. IGD group: higher TMT-B scores than HCs. KYN and KYNA/KYN ratio values intermediate between those of the AUD and HCs.
5	(Kaess et al., 2017) [31]	To investigate differences between patients with IGD and matched controls in everyday stress experience and acute reactivity to experimentally induced stress, using both subjective self-reports and neurobiological measures.	N = 49; male only 15–25 years 24 IGD [18.3 ± 3.3] 25 HCs [19.6 ± 3.4]	A clinical interview by an experienced psychiatrist based on the criteria of the DSM-5 Questionnaires: CSV-S, M.I.N.I-KID 6.0 (for participants in the experimental group under the age of 18), M.I.N.I 5.0 (for adult participants), SCID-N/P (for controls), CTQ, BDI-II, SAS, TICS. Experimental design: Thirty min. heart rate monitor at rest; TSST; Hair samples [cortisol] (basal stress levels); Eight saliva samples (over the experiment); PANAS; VAS.	No group differences on basal hair cortisol, baseline saliva cortisol or heart rate. IGD (vs. HCs): Greater depressive and anxiety symptoms, greater global and chronic stress; Attenuated cortisol response following stress induction; heart rate increases independent of stress induction; lower cortisol; Negative affective stress response significantly and positively correlated with the number of IGD criteria fulfilled, greater psychopathological distress (depression, anxiety, and overall and chronic stress), lower heart rate response (associated with greater cortisol secretion).
6	(Kim et al., 2021) [34]	To search for a quantifiable indicator of addiction in ECG response by investigating various HRV parameters that characterize the particular stress response of game addicts.	N = 23; male only 23 years [±3] 11 GD 12 HC	Questionnaires: CIUS, IAT Game: League of Legends ECG: Various HRV parameters (time-domain parameters: NN interval average, SDNN, SDSD, pNNI50, pNNI20, RMSSD, heart rate average; frequency-domain parameters: LF, HF, LF/HF ratio, LFnu, HFnu, total power, VLF) tested during the game.	No difference of HRV parameters between groups for window sizes of 30 s, 60 s, 90 s, and 120 s after “killing events”. HRV parameters (pNNI20, LF, SDSD, RMSSD, and total power) measured for window sizes of 30 s and 60 s after “killed events” showed a significant difference between the groups.
7	(Kindermann et al., 2016) [32]	To test the hypothesis that playing counter strike as a physiological and physical stressor elevates cortisol levels and due to the presence of threats impairs the intermediate-term memory (ITM) while sports activities as physical stressors elevate cortisol levels but given the absence of a threat, ITM for initially learned facts is supported.	N = 54; male only 16–29 years [18.2 ± 4.32] 18 counter-strike players 18 running group 18 HCs	Experimental design: The LGT-3 learning and memory test; One hour of playing counter; strike/running/spending time outside Recall the previously learned items of the LGT-3 test; Saliva samples [cortisol] (at the beginning and after each phase).	No group differences on baseline cortisol levels. CS group: cortisol decreased due to playing Running group: cortisol increased with time during run Memory retention score: CS-group—−4.28 ± 2.42 points, running-group—0.78 ± 3.82, control group—−1.50 ± 6.11. No correlations between cortisol levels and memory performance.
8	(Koenig et al., 2019) [33]	To assess group differences in hair hormone concentrations comparing young male subjects with IGD and matched healthy controls.	N = 62; male only 15–25 years [18.2 ± 4.32] 31 IGD [18.77 ± 3.01] 31 HCs [20.06 ± 2.94]	Structured clinical interview for IGD diagnosis, developed according to DSM-5 Questionnaires: CSV-S, CTQ, BDI-II, SAS, TICS. M.I.N.I-KID 6.0 (for participants in the experimental group under the age of 18), M.I.N.I 5.0 (for adult participants), and SCID-N/P (for controls). Hair samples for analysis of hair hormones (cortisol, cortisone, testosterone, progesterone, DHEA and corticosterone).	No significant differences on cortisol, cortisone, testosterone, progesterone, DHEA or corticosterone between groups. Greater self-reported anxiety associated with increased cortisol and cortisone. IGD: greater cortisone associated with greater self-reported chronic stress (TICS total).
9	(Porter and Goolkasian, 2019) [36]	To determine if playing video games can induce stress by manipulating video game instructions to evoke threat or challenge appraisals.	N = 148; males 89, females 59 Mean age = 18–38 years [19.92 ± 3.18] 37 challenge-fighting 37 threat-fighting 37 challenge-puzzle 37 threat-puzzle	Experimental design: Participants played a fighting game (Mortal Kombat: Komplete edition) or a puzzle game (Tetris Ultimate). Stress appraisal scales based on Biopsychosocial Model of Challenge and Threat (before and after the game) Cardiovascular responses, heart rate variability and blood pressure (baseline, before and after the game) Positive and negative emotion ratings Self-report questions on Previous Video Game Experience and Game Characteristics	Threat appraisal groups: lower RMSSD during the first 5 min of gameplay; increased RMSSD after the game. Mortal Kombat players: lower RMSSD during the last 5 min of gameplay; increased RMSSD after the game. Interaction effects of game content over time on systolic and diastolic blood pressure. Mortal Kombat players: higher systolic blood pressure post-gameplay. Both Tetris groups: decrease in diastolic blood pressure after receiving instructions; blood pressure increased to baseline levels after the game. Both Mortal Kombat groups: increase in blood pressure after the game.
10	(Roy and Ferguson, 2016) [37]	To examine play style, as well as the effects of gender on cooperative and competitive game play in regards to participant stress levels and heart rates.	N = 100; males 44, females 56 18–25 years 50 competitive group 50 cooperative group	The PASAT test (before, after the game) Game: Lego: Marvel Superheroes (Story mode for cooperative play, Free Play mode for competitive play) Questionnaires: PSM-9 (before, after the game), Experiences survey, Ratings of the confederate Blood Pressure Heart Rate One-way mirror and audio recordings of verbal responses [Physical behaviors and verbal cues monitored without participants awareness]	Heart rate: decline from before to after game play. Blood pressure: systolic pressure decreased in all players from pre to post game play. Cooperative players experienced slightly more decrease in systolic blood pressure than diastolic; NS. Females reported more stress at both pre and post play than males
11	(Snodgrass et al., 2019) [38]	To test whether eudaimonic activity experienced in online play-based environments also manifest in similarly altered immune system regulation.	N = 56; males 36, females 20 Age: 23.79 ± 4.15 13 high involvement gamers Age: 21.69 ± 2.29 43 low involvement gamers Age: 24.42 ± 4.40	Genome-wide transcriptional profiling of dried blood spots (CTRA profile) Questionnaires: MHC-SF, IGDS-SF9, Gaming Involvement, Positive and Negative Gaming Experiences, Offline/Online Social Support, and Gaming-Related Social Norms.	Total mental well-being associated with reduced CTRA expression. Psychobiological relationship between eudaimonia and CTRA appeared most strongly among individuals reporting high levels of positive psychosocial involvement with gaming (i.e., high subjective achievement, social connection, immersion, commitment of time, energy, and effort, and passion/motivation). Low levels of positive involvement with videogames: eudaimonic well-being showed a weaker and nonsignificant negative association with CTRA gene expression

5-HT—5-Hydroxytryptamine; AUD—Alcohol Use Disorder; AUDIT—Alcohol Use Disorder Identification Test; BAI—Beck Anxiety Inventory; BDI—Beck Depression Inventory-II; CANTAB—Cambridge Neuropsychological Test Automated Battery; CD-RISC—Connor–Davidson Resilience Scale; CIUS—Compulsive Internet Use Scale; CSV-S—Scale for the Assessment of Pathological Computer- Gaming; CTRA –Conserved Transcriptional Response To Adversity; CTQ—Childhood Trauma Questionnaire; HCs—Healthy Controls; HRV—heart rate variability; IAT—Internet Addiction Test; IGD—Internet Gaming Disorder; IGDS-SF9—Internet Gaming Disorder Scale, short form of 9-items; ITM—intermediate-term memory; K-CWST—The Korean Color–Word Stroop Test; KYN—Kynurenine; KYNA— Kynurenic Acid; M.I.N.I. 5.0—Mini-7 International Neuropsychiatric Interview; M.I.N.I-KID 6.0—Mini-International Neuropsychiatric Interview for Children and Adolescents; MHC-SF—Mental Health Continuum-Short Form; NS—Not Significant; PANAS—short version of the Positive and Negative Affect Schedule; PNGE-42—Positive and Negative Gaming Experiences Scale; PSM-9—Psychological Stress Measures Questionnaire; PWI—Psychosocial Well-being Index; SAS—the Zung Self-Rating Anxiety Scale; SCID-N/P—the Structured Clinical Interview for DSM-IV-TR Non-Patient edition; SSP—Spatial Span; SWM—Spatial Working Memory; TICS—Trier Inventory for Chronic Stress; TICS—Trier Inventory for Chronic Stress; TMT—Trail-Making Test; TRP—Tryptophan; TSST—Trier Social Stress Test; VAS—visual analogue scales; WAIS-IV—The Korean version of the Wechsler Adult Intelligence Scale-IV; Y-IAT—Young’s Internet Addiction Test.

### 2.4. Measurements of Stress Response Related to Gaming

Multiple approaches were used to measure stress reactivity in gaming research. These include: (1) blood pressure cuff/heart rate monitor (measured in 4 studies) [34,36,37,39]; (2) EEG (measured in 3 studies) [28,29,30]; (3) saliva samples collected for cortisol and α-amylase (measured in 5 studies) [28,29,30,31,32]; (4) hair samples for analysis of hair hormones (cortisol, cortisone, testosterone, progesterone, DHEA, and corticosterone) (measured in 2 studies) [31,33]; (5) analysis of Kynurenine Pathway Metabolites: TRP, 5-HT, KYN, and KYNA levels in serum samples (measured in 2 studies) [35,38].

## 3. Discussion

### 3.1. Neurobiological Evidence on Stress-Related Gaming Research

Some of the research specifically assessed the acute effect of gaming on stress response activity, while others measured the long-term gaming-related effect on stress. The findings of the research included in this review were grouped by their specific primary measurements.

### 3.2. Blood Pressure (BP) and Heart Rate (HR)

BP and HR are sensitive to changes in autonomic balance, and stress could lead to excess sympathoadrenal activation, influencing BP and HR [39]. The findings of gaming research on BP and HR have been inconsistent. In a study assessing the effect of gaming on acute stress, a lab-based stressor was introduced to the participants prior to gaming. In line with reduced self-reported stress, both heart rate and blood pressure were lower after game play [37]. The authors argued that game play not only provides a break to players who can escape from stressful events in real life, but also that a sense of control and autonomy gained through gaming could contribute to stress relief. Similarly, a study that examined the cardiovascular stress response to gaming failed to identify any significant game-related change in BP and HR, suggesting that video games do not act as mental stressors and are unable to induce stress [36]. Nevertheless, specific instructions for game playing and type of game could lead to differences in cardiovascular stress responses; for example, when participants were told that they would be evaluated based on their performance of gaming, increased negative emotion ratings and decreased heart rate variability were observed. Compared to the puzzle game players, fighting game players showed increased blood pressure and decreased heart rate variability and also reported higher positive emotion ratings [36]. In contrast, a modest stress response associated with video gaming has also been reported: playing a video game for 1 h resulted in greater systolic blood pressure (SBP), heart rate, and feelings of stress [40].

### 3.3. EEG and Saliva Samples

EEG signals containing information about mental states might be a helpful tool to measure changes in brain activity patterns caused by stress [41,42]. EEG asymmetry index, power, coherence, and other features were widely investigated in stress studies, with the alpha asymmetry index and greater frontal right alpha activity being a consistent and robust stress-related feature reflecting emotional arousal [42,43].

Salivary cortisol is routinely used as a standard to measure stress response and is appreciated as a marker of HPA axis activity [44], with increased cortisol secretion in response to stress. Cortisol can interact with the brain’s reward system and might be involved in addictions by contributing to the reinforcing effects of substances such as alcohol [45]. Such results led to the investigation of dysregulated HPA axis responses to a stressor in non-substance addictive disorders as well; however, considering IGD findings are mixed [26].

Only several studies addressed the EEG in relation to gaming in conjunction with saliva sample analysis. EEG was recorded during the play of a football match using a 14-channel EMOTIV system, and saliva samples were collected before and after the game. The results show that salivary cortisol concentration was significantly decreased after gaming, and the power of the EEG signal was increased in most of the electrodes in lower bands (3 Hz) and higher frequency bands (15 Hz), but decreased in the occipital lobe (in 6 and 10 Hz) and in the frontal lobe (in 15 and 18 Hz) [28]. Unfortunately, the authors did not elaborate on the implication of brain wave change in stress relief. In contrast, opposite results were also reported—the stress marker (salivary cortisol) and the fear marker (α-amylase) levels increased after playing the brain teaser game [30].

Furthermore, the specific content-related effect of video games was also evaluated [29]. EEG and the concentrations of cortisol and α-amylase were measured prior to and following playing four different types of games, respectively, including the Runner game, Excitement game, Fear game, and Puzzle game. It has been found that the concentration of α-amylase and cortisol increased significantly after playing the Fear game, Runner game, and Excitement game but decreased after playing the Puzzle game. The secretion of the salivary enzyme (α-amylase) is a reliable measure of the activity of the sympathetic nervous system, and its increase is often associated with stress-induced fear, which can happen quickly within a few seconds [46]. On the other hand, cortisol is considered the most important stress hormone secreted by the adrenal cortex of humans, and its effect on mental function could be modulated by the duration and intensity of stress. Acute stress enhances mental abilities, while chronic stress leads to dysfunction. Thus, reduction in α-amylase and cortisol in puzzle games may indicate the deactivation of the stress system due to the mental demand required for problem solving in puzzle games. In contrast, the Runner game, Excitement game, and Fear game increase the stress response, which may lead to impairment in cognitive function in the long run [29]. In line with this, impaired memory after a violent game was also reported by others; however, cortisol levels were decreased in this case [32].

### 3.4. Hair Sample

In contrast, a study using hair samples for the analysis of cortisol, cortisone, testosterone, progesterone, dehydroepiandrosterone (DHEA), and corticosterone reported negative results, showing no significant difference in basal HPA axis functioning between individuals with IGD and healthy controls [33]. However, a lack of change in measures of hair cortisol does not exclude the possibility of altered neurobiological stress reactivity to acute stress in IGD. Research shows that although patients with IGD show no higher basal HPA axis activity than health controls, they exhibit increased levels of subjective stress, negative affect, and heart rate, as well as transiently decreased levels of cortisol during a standardised laboratory stress task, and this greater negative stress response was strongly correlated with IGD severity [31].

### 3.5. Blood Sample

The close link between stress and the pathophysiology of addictive disorders is well established. Thus, some researchers have compared the stress-related biological profile of individuals with Internet gaming disorder (IGD) with that of those with substance use disorder. For example, a study examined the differences in stress level and serum levels of tryptophan (TRP), 5-hydroxytryptamine or serotonin (5-HT), kynurenine (KYN), and kynurenine acid (KYNA) between young adults with IGD, those with alcohol use disorder (AUD), and health control [35]. The KYN pathway is one of the TRP metabolism pathways, which is initiated by the enzyme indoleamine 2,3-dioxygenase (IDO) and the 5-HT, and alterations of TRP level metabolism could be caused by either stress and/or immune system activation. The results demonstrated that the KYN levels and KYNA/KYN ratios for the IGD group were intermediate between those of the AUD group and healthy controls, showing increased KYN levels and KYN/TRP ratios and decreased KYNA levels and KYNA/KYN ratios relative to the healthy controls; nevertheless, the level of TRP was not different between groups [35]. Furthermore, higher levels of stress, lower resilience, and impaired executive functions were found in both addictive disorder groups compared to the health controls. These findings were considered to be an indication of vulnerable neuronal networks associated with addictive disorders, which could be caused by great exposure to stress.

In line with this, an association between increased social wellbeing and reduced expression of a stress-induced gene profile, so-called “conserved transcriptional response to adversity” (CTRA), has been found in individuals experiencing positive psychosocial effects from gaming, such as motivation for achievement, socialisation, and immersion via gaming, but this association has become weaker and non-significant in those with a low level of positive involvement in gaming. It appears that the effects of gaming could be different from one person to another due to individual differences. The authors have proposed the “rich-get-richer” and “poor-get-poorer” effects in the context of gaming and argued that videogaming could be considered a therapeutic device primarily for psychosocially healthy individuals [38].

## 4. What Are Implications of Gaming-Related Stress Research?

There is dynamism and complexity in the immune system and addiction, such as large interindividual variations of inflammation, a history of addiction, medical or psychiatric symptoms associated with addiction, etc. Video game addiction, conceptualised as a non-chemical addiction, not only shares many key addiction symptoms, such as loss of control, craving, and mood regulation, but also affects the brain, activating the brain’s reward system in a similar way as substance use disorder. Without the confounding problems of polydrug use and drug effects, it may provide a competing way to investigate addiction itself.

Based on the research evidence included in this review, it appears that it remains unclear whether gaming helps to reduce stress or not. Even in the case when the authors acknowledged that a reduction in stress after gaming has been observed, a function of time on stress reduction could not be ruled out, and it is unclear how gaming compares to other stress-reducing activities [37]. Nevertheless, gaming could induce a physiological stress response, such as increased blood pressure paired with a decreased heart rate [36], reduced cardiac coherence [47], and increased salivary cortisol [29]. The extent of the altered physiological response might be related to either the actual content and type of game or the intensity of game playing. In some cases, the content and type of game could be considered stressors of varying degrees, while the intensity of gaming may reflect the severity of problematic gaming.

Furthermore, some of the physiological stress response induced by gaming might be related to biological correlates, as evidence showed differences in gene expression profiles between functional gamers and individuals with IGD. The association between problematic internet use and reduced self-reported immune function that is independent of the impact of comorbidities such as depression, isolation, and anxiety has been reported [48]. For example, the conserved transcriptional response to adversity (CTRA), a leukocyte gene expression profile activated by chronic stress. It has been found that persons with more frequent positive gaming experiences are less likely to manifest the elevated CTRA genomic profile compared to those with IGD [49]. In line with this, abnormal neurobiology in the brain regions related to impulse control, reward processing, and somatic memory, such as the orbitofrontal cortex (OFC), striatum, and sensory regions, has been found in people who are excessive internet game users. Compared to normal game users, those excessive internet game users show greater impulsiveness, and the intensity of game play is positively correlated to impulsiveness [50]. It has been suggested that the psychological and neural mechanisms underlying other types of impulse control disorders and substance/non-substance-related addiction may be shared by game overuse [50].

This scoping review comes with limitations that need to be acknowledged. The majority of the study samples included in this review were male, and this would compromise the generalizability of our results to the general population. Furthermore, video game types are also quite heterogeneous (e.g., competitive action game vs. puzzle game), and the results presented in this review primarily focused on gaming in general rather than the specific type of game-related effect. We only included the research published in English in the last ten years, and given the relatively high prevalence of gaming in Asian countries, we might miss out on some invaluable evidence published in other languages.

In conclusion, gaming could stimulate a stress-like physiological response, and the direction of this response might be modulated by an individual’s biological profile, history of gaming, and content of gaming. To date, understanding the health impact of gaming is still in its infancy. By acknowledging the positive effect of gaming on cognition and a person’s life, its potential harm should not be ignored, and this should call for more biologically-based research to reveal what factors lead to individual differences in the development and maintenance of problematic behaviours.

## Data Availability

Not applicable.
